# European recommendations and quality assurance for cytogenomic analysis of haematological neoplasms: reponse to the comments from the Francophone Group of Hematological Cytogenetics (GFCH)

**DOI:** 10.1038/s41375-020-0736-x

**Published:** 2020-02-07

**Authors:** K. A. Rack, E. van den Berg, C. Haferlach, H. B. Beverloo, D. Costa, B. Espinet, N. Foot, S. Jeffries, K. Martin, S. O’Connor, J. Schoumans, P. Talley, N. Telford, S. Stioui, Z. Zemanova, R. J. Hastings

**Affiliations:** 1 GenQA, John Radcliffe Hospital, Oxford University Hospitals NHS Trust, Oxford, UK; 2 Department of Genetics, University Medical Center Groningen, University of Groningen, Groningen, The Netherlands; 3 MLL-Munich Leukemia Laboratory, Munich, Germany; 4 Department of Clinical Genetics, Erasmus MC, University Medical Center, Rotterdam, The Netherlands; 5 Hematopathology Section, Hospital Clinic, Barcelona, Spain; 6 Laboratori de Citogenètica Molecular, Servei de Patologia, Grup de Recerca, Translacional en Neoplàsies Hematològiques, Cancer Research Program, imim-Hospital del Mar, Barcelona, Spain; 7 Viapath Genetics Laboratories, Guys Hospital, London, UK; 8 West Midlands Regional Genetics Laboratory, Birmingham Women’s Hospital, Birmingham, UK; 9 Department of Cytogenetics, Nottingham University Hospital, Nottingham, UK; 10 Haematological Malignancy Diagnostic Service, St James’s University Hospital, Leeds, UK; 11 Oncogénomique laboratory, Hematology Department, Lausanne University Hospital, Vaudois, Switzerland; 12 Oncology Cytogenetics Service, The Christie NHS Foundation Trust, Manchester, UK; 13 Laboratorio di Citogenetica e genetica moleculaire, Laboratorio Analisi, Humanitas Research Hospital, Rozzano, Milan, Italy; 14 Center of Oncocytogenetics, Institute of Clinical Biochemistry and Laboratory Diagnostics, General University Hospital and First Faculty of Medicine, Charles University in Prague, Prague, Czech Republic

**Keywords:** Genomics, Haematological cancer

The authors would like to thank the GFCH for their commentary on the recommendations for cytogenomic testing [1]. We are pleased to note that they are in agreement for the majority of aspects of our recommendations and appreciate where the GFCH have expanded or provided clarification to our statements.

In particular we were pleased that the GFCH concur with the authors of the importance and value of chromosome-banding analysis in the diagnostic pathway of haematological neoplasms as well as the complementary nature of different testing strategies. We note that the GFCH has a few concerns and these are addressed individually below.

In replying to their comments, it is important to restate that the recommendation document is a consensus document of working practices for cytogenomic testing in a number of different European countries and describes the minimum testing required, and that national policies should be taken into consideration [2]. We acknowledge that more extensive testing is undertaken in some countries particularly relating to the number of metaphases analysed and the pathological entities tested by chromosome-banding analysis. The aim of the recommendations was to provide the practical advice to help laboratories prioritise and rationalise cytogenomic testing where health care resources are restricted and where the extent of testing is limited by testing reimbursement.

Choice of sample: We agree with this recommendation and thank them for the extra clarification provided to our statement regarding the use of peripheral blood samples for chromosome-banding analysis in this section.

For cell culture of AML the authors agree that these rearrangements can be detected in 24 h cultures. The authors state that a 48 h culture should be considered but this was not intended to be a requirement. This observation referred mainly to historic data of t(15;17) cases when FISH or RT-PCR were not widely available.

Choromosome banding analysis: We agree that the full ISCN 2016 definition of a clone includes the example cited but this is also covered by the statement provided in our paper.

We agree that ideally 20 metaphases would be analysed regardless of the result, and indeed are aware that many laboratories do systematically analyse this number. However, a minimum of ten metaphases are considered acceptable when an abnormal clone is detected, unless there is suspicion of clonal evolution (e.g., one metaphase with additional abnormality) where a more extensive analysis should be performed.

Recommended testing: We state that chromosome-banding analysis is mandatory for ALL in Table 2 [2]. However, for information, we refer to the publication of the International Berlin–Frankfurt–Münster study group on recommendations for the detection of prognostically relevant genetic abnormalities in childhood B-cell precursor acute lymphoblastic leukaemia [3]. The vast majority of laboratories undertake chromosome analysis of childhood B-cell ALL but our experience through EQA has also shown that some laboratories are only following this strategy when cases require risk stratification. Of note, for the new childhood ALLTogether trial karyotyping is not mandatory in all cases provided sufficient extensive first line testing using FISH, PCR, and SNP-array is performed.

FISH in AML: Regarding *NUP98* in AML, the authors are in agreement with this approach. We recognise that the text should have stated 17 months-18 years not 5 years.

Waldenström macrogobulinemia (WM): The authors consider that cytogenomic testing, including chromosome analysis, for recurrent abnormalities should be undertaken as the detection of disease-specific recurrent abnormalities can be beneficial in a differential diagnosis case. Many laboratories would not undertake routine chromosome analysis for all WM cases although we recognise that country-specific guidelines may recommend this.

High grade B-cell lymphomas: We are in concordance with the overall choice of probes to be used for this disease entity and agree that as *MYC* breakpoints are located across a large genomic region the choice of adequate FISH probe(s) is paramount. It is therefore important that laboratories understand the limitations of FISH probes when they select which probes to use.

Concerning chromosome analysis, many laboratories do not undertake routine chromosome analysis of high grade B-cell lymphoma where FISH is the priority testing for these cases. We agree that karyotyping is useful, however, this is not always possible. In addition, when karyotyping is performed, this is also often supplemented by FISH analysis. Many laboratories no longer undertake chromosome analysis but instead perform FISH on FFPE sections to detect the most significant abnormalities.

Reporting time: We are aware that a large number of examinations are requested, and that laboratories can find it difficult to adhere to recommended turnaround times but it is important that laboratories organise the work flow so that reporting times can be met. Concerning prioritisation of testing, we recommend that this is assigned according to clinical need as reporting results in follow-up samples can also be urgent since targeted therapy is frequently used in a relapse or emerging relapse setting.

In summary, the authors are grateful for the response by the GFCH to our recommendations as this has enabled us to clarify certain points and address a few omissions. In addition, both the recommendations and the GFCH underline the importance of cytogenetics and a combined approach to testing. Finally, the authors recognise that different countries will have different approaches to ensure that full testing is addressed and adoption of these recommendations, together with this clarification, will assist in further harmonisation of genetic testing.
